# Political Ideology and Trust in Government Health Agencies for Cancer Information

**DOI:** 10.1001/jamanetworkopen.2023.41191

**Published:** 2023-11-03

**Authors:** Onyema G. Chido-Amajuoyi, Henry K. Onyeaka, Itunu O. Sokale, Somto Nwani, Carlos H. Barcenas, Hermioni L. Amonoo, Sanjay Shete

**Affiliations:** 1 Department of Epidemiology, The University of Texas MD Anderson Cancer Center, Houston; 2 Department of Internal Medicine, Texas A&M School of Medicine/Christus Health, Longview; 3 Department of Psychiatry, Harvard Medical School, Boston, Massachusetts; 4 Department of Psychiatry, Massachusetts General Hospital, Boston, Massachusetts; 5 Department of Medicine, Baylor College of Medicine, Houston, Texas; 6 Faculty of Pharmaceutical Sciences, University of Nigeria, Enugu, Nigeria; 7 Department of Breast Medical Oncology, The University of Texas MD Anderson Cancer Center, Houston; 8 Department of Psychiatry, Brigham and Women’s Hospital, Boston, Massachusetts; 9 Department of Psychosocial Oncology and Palliative Care, Dana-Farber Cancer Institute, Boston, Massachusetts; 10 Department of Biostatistics, The University of Texas MD Anderson Cancer Center, Houston; 11 Division of Cancer Prevention and Population Sciences, The University of Texas MD Anderson Cancer Center, Houston

## Abstract

This cross-sectional study assesses the association between political ideology and trust in government health agencies for cancer information.

## Introduction

Government health agencies have traditionally been considered highly credible sources of health information.^1^ However, agencies, such as the US Centers for Disease Control and Prevention and the US Food and Drug Administration, have witnessed decreases in levels of trust^2^ amid increasing credibility concerns.^1^ Inconsistent messaging, misinformation, and political division during the COVID-19 pandemic further exacerbated the eroding public trust in government health agencies^1,2,3^ for health-related information, including cancer information.^4^

While political ideology has been shown to be broadly associated with trust in government,^5^ its association with trust in government health agencies is not well established. Therefore, this study aims to examine political ideology and trust in government health agencies for cancer information.

## Methods

We examined data from the National Cancer Institute Health Information National Trends Survey (HINTS) 5 Cycle 4, conducted from February 24 through June 15, 2020. Data were analyzed from January 30 to April 5, 2023. HINTS is a nationally representative survey of noninstitutionalized civilian US adults with a survey response rate of 36.7%. HINTS data are publicly available and deidentified. Therefore, our study is exempt from institutional review board review per the Common Rule (45 CFR §46). This study followed the STROBE reporting guideline. The outcome of interest was the level of trust in government health agencies as a source of cancer information, and the exposure variable was political ideology. Among the survey respondents, more than 90% responded to the specific questions on trust in government health agencies as a cancer information source and political ideology.

Multivariable logistic regression analysis was used to assess political ideology and trust in the various sources of cancer information. Analysis was adjusted for age, sex, race and ethnicity, educational level, household income, having a regular health care professional, presence of comorbidities, and rural-urban residence. Race was assessed as part of the sociodemographic characteristics of study participants and was self-reported. The statistical significance level was defined as 2-sided *P* < .05. Statistical analyses were performed using Stata 17.0 (StataCorp LLC).

## Results

The overall sample of 3254 respondents included 1794 women (50.1%); 1965 White (64.9%); 396 Black or African American (10.7%); 499 Hispanic (16.6%), and 249 other (7.8%) individuals; 2895 respondents (88.0%) were urban residents. A total of 78.1% of respondents reported having some or a lot of trust in government health agencies as a cancer information source (Table 1). For political ideology, 37.2% of respondents identified as being moderate, 29.5% as liberal, and 33.3% as conservative. In the adjusted logistic regression models (Table 2), conservative viewpoints were associated with lower odds of having some or a lot of trust in government health agencies for cancer information compared with having liberal viewpoints (adjusted odds ratio, 0.46; 95% CI, 0.30-0.71) and compared with having moderate viewpoints (adjusted odds ratio, 0.69; 95% CI, 0.50-0.94).

**Table 1.** Prevalence of Trust In Government Health Agencies for Cancer Information by Sociodemographic Characteristics and Political Ideology (N = 3254)^a^

Sample characteristic	Total, No. (weighted %)	Trust in government health agencies, weighted % (95% CI) [No.]		*P* value
		Little/not at all (n = 707)	Some/a lot (n = 2547)	
Sex				
Female	1794 (50.1)	21.4 (18.6-24.6) [389]	78.6 (75.4-81.4) [1405]	.86
Male	1338 (49.9)	21.9 (18.0-26.6) [286]	78.1 (74.0-81.8) [1052]	
Age group, y				
18-34	451 (27.5)	17.1 (11.8-24.1) [78]	82.9 (75.9-88.2) [373]	.10
35-49	635 (26.4)	22.9 (18.2-28.5) [122]	77.0 (71.5-81.8) [513]	
50-64	990 (27.3)	21.4 (17.6-25.8) [223]	78.6 (74.2-82.4) [767]	
≥65	1121(18.8)	27.0 (22.9-31.4) [268]	73.0 (68.6-77.1) [853]	
Educational level				
High school or less	739 (27.9)	31.5 (26.3-37.2) [216]	68.5 (62.8-73.7) [523]	.001
Some college	953 (40.0)	19.7 (15.5-24.5) [212]	80.0 (75.5-84.5) [741]	
College graduate or more	1550 (32.1)	16.1 (13.3-19.4) [276]	83.9 (80.6-86.7) [1274]	
Annual household income, US $				
<20 000	473 (13.3)	19.9 (14.6-26.4) [127]	80.1 (73.6-85.4) [346]	.16
20 000-34 999	389 (10.7)	24.8 (19.6-30.8) [103]	75.2 (69.3-80.4) [286]	
35 000-49 999	411 (12.1)	28.3 (19.8-38.7) [102]	71.7 (61.3-80.2) [309]	
50 000-74 999	555 (19.1)	23.8 (17.2-32.0) [117]	76.2 (68.0-82.8) [438]	
≥75 000	1263 (44.8)	18.9 (15.6-22.7) [217]	81.1 (77.3-84.4) [1046]	
Race and ethnicity^b^				
Black or African American	396 (10.7)	26.1 (19.3-34.3) [87]	73.9 (65.7-80.7) [309]	.42
Hispanic	499 (16.6)	17.8 (12.0-25.5) [93]	82.2 (74.5-88.0) [406]	
White	1965 (64.9)	21.9 (18.9-25.1) [435]	78.1 (74.9-81.1) [1530]	
Other^c^	249 (7.8)	21.7 (14.2-31.8) [47]	78.3 (68.2-85.8) [202]	
Residence				
Urban	2895 (88.0)	21.5 (19.0-24.3) [611]	78.5 (75.7-81.0) [2284]	.44
Rural	359 (12.0)	24.3 (18.1-31.7) [96]	75.7 (68.3-81.9) [263]	
Having a regular health care clinician				
No	946 (37.1)	25.9 (21.6-30.8) [242]	74.1 (69.2-78.4) [704]	.01
Yes	2269 (62.9)	19.5 (17.0-22.2) [457]	80.5 (77.8-83.0) [1812]	
Comorbidity				
None	1264 (48.6)	20.0 (16.6-24.0) [264]	80. 0 (76.0-83.4) [1000]	.18
At least 1	1960 (51.4)	23.4 (20.3-26.7) [432]	76.6 (73.3-80.0) [1528]	
Political ideology^d^				
Conservative	1129 (33.3)	27.4 (22.9-32.3) [291]	72.6 (67.7-77.0) [838]	.001
Moderate	1130 (37.2)	21.7 (17.9-26.0) [253]	78.3 (74.0-82.0) [877]	
Liberal	995 (29.5)	15.8 (12.6-19.6) [163]	84.2 (80.4-87.4) [832]	

^a^ Participants were asked to assess their trust in government health organizations as sources of cancer information with the following prompt: “In general, how much would you trust information about cancer from government health agencies?” Response options included “a lot,” “some,” “a little,” and “not at all.” A lot and some were collapsed into a single response category, and a little and not at all were also collapsed into a single response category.

^b^ Race was assessed as part of the sociodemographic characteristics of study participants and was self-reported.

^c^ No further break down of the other category was available from the National Cancer Institute Health Information National Trends Survey.

^d^ Political ideology was derived with the survey question, “Thinking about politics these days, how would you describe your own political viewpoint?” Responses were categorized into 3 groups. Very liberal, liberal, and somewhat liberal were categorized “liberal,” and very conservative, conservative, and somewhat conservative were categorized “conservative.” The final category was “moderate.”

**Table 2.** Trust in Government Health Agencies for Cancer Information (n = 2780)^a^

Political ideology^b^	Level of trust, AOR (95% CI)^c^	
	Little/not at all	Some/a lot
Conservative (compared with liberal [reference])	1 [Reference]	0.46 (0.30-0.71)
Conservative (compared with moderate [reference])	1 [Reference]	0.69 (0.50-0.94)
Liberal (compared with moderate [reference])	1 [Reference]	1.48 (0.96-2.27)

Abbreviation: AOR, adjusted odds ratio.

^a^ Participants were asked to assess their trust in government health organizations as sources of cancer information with the following prompt: “In general, how much would you trust information about cancer from government health agencies?” Response options included “a lot,” “some,” “a little,” and “not at all.” A lot and some were collapsed into a single response category, and a little and not at all were also collapsed into a single response category.

^b^ Political ideology was derived with the survey question, “Thinking about politics these days, how would you describe your own political viewpoint?” Responses were categorized into 3 groups. Very liberal, liberal, and somewhat liberal were categorized “liberal,” and very conservative, conservative, and somewhat conservative were categorized “conservative.” The final category was “moderate.”

^c^ Adjusted for age, sex, race and ethnicity, educational level, household income, having a regular health care clinician, presence of comorbidities, and rural-urban residence.

## Discussion

In this nationally representative cross-sectional study of US adults, findings suggest that conservative viewpoints were associated with lower trust in government health agencies for obtaining cancer information. This finding has substantial implications, affecting various aspects of cancer prevention and treatment. First is potential adherence to cancer prevention practices, including cancer screening guidelines. Second, those with lower levels of trust in information from health agencies may face challenges in making well-informed decisions about their treatment, which could affect their overall health care experience and outcomes.

Prior research indicates that conservative viewpoints are associated with skepticism of government institutions.^6^ Mirroring our findings, a study^6^ found policy liberalism enhanced trust in the US Centers for Disease Control and Prevention and the World Health Organization and adherence to their guidelines during the COVID-19 pandemic. Study findings are limited by the possibility of low-response bias associated with HINTS and other population-based surveys and the potential for residual confounding.

Potential strategies to build trust in government health agencies include tailoring health communication to resonate with individuals across the political spectrum. Additional studies are warranted to further our understanding of the intricacies underlying trust in government.

## Notes

Supplement.
Data Sharing Statement
