# Supplementary material for: Political Ideology and Trust in Government Health Agencies for Cancer Information
Source: JAMA Netw Open. 2023 Nov 3;6(11):e2341191. doi: 10.1001/jamanetworkopen.2023.41191 (PMC10625042; doi:10.1001/jamanetworkopen.2023.41191)
Supplement: Supplement. — Data Sharing Statement [file jamanetwopen-e2341191-s001.pdf]

Data Sharing Statement

Chido-Amajuoyi. Political Ideology and Trust in Government Health Agencies for Cancer Information. *JAMA Netw Open*. Published November 03, 2023. doi:10.1001/jamanetworkopen.2023.41191

Data
Data available: No
